# Association between night eating frequency and thyroid function and sensitivity: a cross-sectional study from the NHANES database

**DOI:** 10.3389/fendo.2024.1489459

**Published:** 2024-12-23

**Authors:** Yanhao Zhang, Songbai Zhou, Shiguang Liu, Youlin Wang, Houyong Zhou, Jiao Wang, Ling Wang, Xiaosong Wang

**Affiliations:** ^1^ Department of Clinical Laboratory, Xichang People’s Hospital, Xichang, China; ^2^ Department of Thoracic Surgery, Xichang People’s Hospital, Xichang, China; ^3^ Department of Gastroenterology, Xichang People’s Hospital, Xichang, China; ^4^ Department of Gastrointestinal Surgery, General Surgery, Xichang People’s Hospital, Xichang, China

**Keywords:** thyroid function, thyroid sensitivity, circadian rhythms, night eating, cross-sectional study 31 32

## Abstract

Thyroid function is closely linked to circadian rhythms, but the relationship between the frequency of night eating and thyroid function remains unclear. Our study aimed to investigate the association between night eating frequency and its impact on thyroid function and sensitivity. This study included 6093 participants from the U.S. National Health and Nutrition Examination Survey (2007–2012). Night eating behavior was assessed through 24-hour dietary recall, with night eating frequency calculated on the basis of food intake between 10:00 PM and 4:00 AM. The thyroid hormone indices examined included T3, T4, FT3, FT4, TSH, TGA, Tg, and TPOAb, whereas thyroid hormone sensitivity was assessed via indices such as the FT3/FT4, TSHI, TT4RI, and TFQI. The associations between night eating frequency and thyroid function were analyzed via weighted univariate and multivariate linear regression analyses. Subgroup analyses and interaction test analyses were also employed to test this correlation. Compared with individuals who did not eat at night, those who ate more frequently at night had higher levels of Tg (OR 1.223 [95% CI 1.048, 1.429], p trend=0.015) but lower levels of T3 (OR 0.728 [95% CI 0.611, 0.868], p trend=0.235) and TPOAb (OR 0.728 [95% CI 0.611, 0.868], p trend=0.235). Subgroup analysis indicated that this association between Tg and night eating was stronger in the DM group (Tg: OR 1.49 [95% CI 1.15, 1.93]), p interaction=0.022) and that the association between TPOAb and night eating was stronger in the group without DM (TPOAb: OR 0.9 [95% CI 0.82, 0.97]), p interaction=0.003). Our findings suggest a significant association between night eating frequency and thyroid function. However, no statistically significant differences were found in thyroid sensitivity based on night eating frequency. Despite these findings, the hormone fluctuations observed were within normal clinical ranges. Further rigorously designed studies are needed to establish a causal relationship between night eating frequency and thyroid function.

## 1 Introduction

Thyroglobulin (Tg) is synthesized and stored within the thyroid follicular lumen (1). When the body requires thyroid hormone synthesis, thyroglobulin undergoes iodination and enzymatic digestion, leading to the production of thyroxine (T4) and triiodothyronine (T3). Thyroid peroxidase (TPO) is the key enzyme responsible for this biosynthesis (2). T3 and T4 are essential for human growth, development, and metabolism; they act on multiple target organs and play crucial roles in thermogenesis, protein synthesis, glucose metabolism, and nervous system development (3). The biologically active forms of these hormones are free T3 (FT3) and free T4 (FT4). The secretion of thyroid hormones, which includes thyroid-stimulating hormone (TSH) and thyrotropin-releasing hormone (TRH), is regulated by the hypothalamic–pituitary–thyroid axis. In addition to primary hormones, several calculated indices are commonly used to assess thyroid sensitivity: FT3/FT4 for evaluating peripheral thyroid hormone sensitivity and the thyroid feedback quantile-based index (TFQI), TSH index (TSHI), and thyrotroph T4 resistance index (TT4RI) for assessing central thyroid hormone sensitivity (4–6).

Research on circadian rhythms indicates that in metabolically healthy adults, various metabolic cycles, insulin secretion and sensitivity, and energy expenditure follow a rhythmic pattern (7). Maintaining alignment with circadian rhythms in sleep and diet can reduce the risk of diseases (8). On the other hand, night eating has been shown to impair glucose tolerance (9), trigger mental disorders (10), and accelerate weight gain (11) by disrupting these circadian rhythms. The consumption of foods, such as high-fat, high-sugar, high-salt snacks at night, can further increase total cholesterol and low-density lipoprotein (LDL) cholesterol levels while reducing fat oxidation (12, 13). Therefore, night eating may disrupt the balance of endocrine hormone secretion.

Several studies have demonstrated that disruptions in circadian rhythms can interfere with TSH secretion, thereby affecting thyroid function (14). However, no studies have specifically examined the impact of night eating frequency on thyroid indicators. To address this gap, our study explored the relationships between night eating frequency and both thyroid function and thyroid sensitivity.

## 2 Materials and methods

### 2.1 Study population

**Figure 1:**
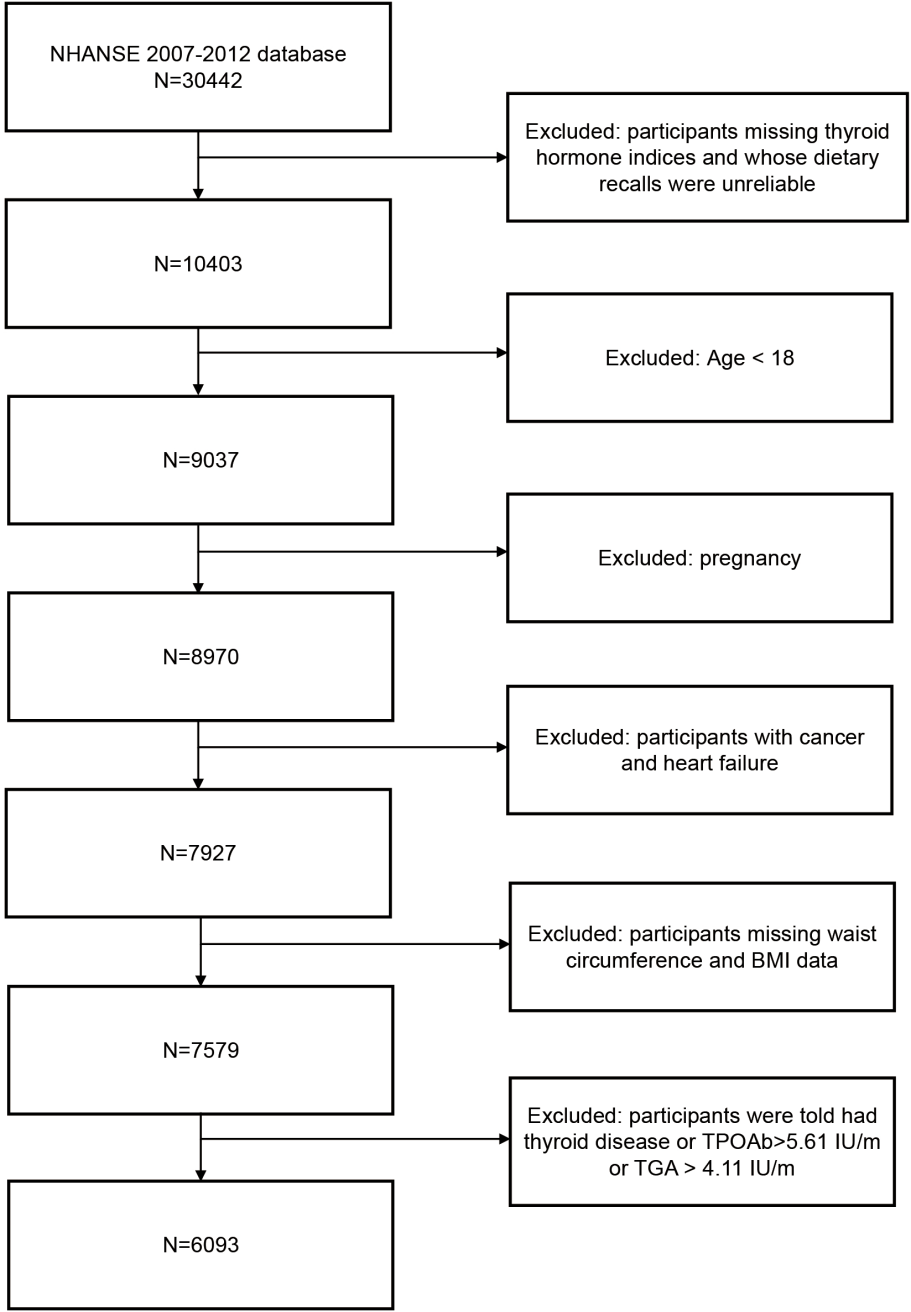
Flowchart of the study population.

The data from 2007–2008, 2009–2010, and 2011–2012 were used for this study (n = 30442). We excluded 20039 individuals whose dietary recall data were unreliable and thyroid hormone indices were missing, 1366 participants younger than 18 years of age, 67 pregnant participants, 1043 participants with heart failure and cancer, 348 participants whose BMI and waist circumference data were missing, and 348 participants with thyroid disease. Overall, our sample consisted of 6093 participants (**Figure 1**).

### 2.2 Dietary assessment

The baseline dietary intake data from 2007-2012 were gathered from the initial 24-hour dietary recall interview. This first recall was conducted in person by trained personnel at the NHANES mobile examination centers. Standardized protocols and measurement tools were used to assess the volume and dimensions of the food. During the interviews, the participants were asked to provide details about the quantity and timing of each food and beverage they consumed. The nutrient values were calculated via the Food and Nutrient Database for Dietary Studies (FNDDS).

### 2.3 Main exposures

The main exposure was the frequency of night eating. Night eating was defined as food consumption between 10:00 PM and 4:00 AM, on the basis of the natural light cycle rhythm in this study. The frequency of night eating was categorized into three groups: “No night eating”, “One time”, and “Two times and over”.

### 2.4 Thyroid function and sensitivity

In this study, the levels of TT3, FT3, and TT4 were measured via a competitive binding immunoenzymatic assay. Free T4 was determined through a two-step enzyme immunoassay. Sensitive human thyroid-stimulating hormone was detected via a two-site “sandwich” immunoenzyme test. TgAb (TGA) and TPOAb (IU/mL) levels were evaluated via a sequential two-step immunoenzymatic “sandwich” assay, whereas Tg levels (ng/ml) were measured via a simultaneous one-step “sandwich” assay. Detailed instructions for sample collection and processing are discussed in the NHANES Laboratory/Medical Technologists Procedures Manual (LPM). The following is the calculation method for sensitivity to thyroid hormone indices:

FT3/FT4 is achieved by FT3/FT4 ratio=FT3/FT4, TSHI is achieved by TSHI= lnTSH+0.1345*FT4, TT4RI is achieved by TT4RI=FT4*TSH, TFQI is achieved by TFQI = cumulative distribution function (cdfFT4) – (1 – cdfTSH).

### 2.5 Covariates

The covariates included age (years), sex (male/female), race/ethnicity (Mexican American/non-Hispanic Black/non-Hispanic White/other), education (below high school/high school/above high school), income (poor/not poor), smoking status (never/former/now), drinking status (yes/no), body mass index (<25/25~29/≥30), physical activity (yes/no), hours of sleep (<6h/6~8 h/>8 h), hyperlipidemia (yes/no), hypertension (yes/no), diabetes (DM, yes/no), obesity (yes/no), abdominal obesity (Aobesity,yes/no), UIC (urinary iodine concentration, <100 (deficient), 100-299 (normal), ≥300 µg/L (excess)) (15), glycohemoglobin (%), TG (mmol/L), Ur (mmol/L), Cr (µmol/L), TC (mg/dL), LDL (mg/dl), HDL (mg/dl), neutrophil–lymphocyte ratio (NLR). Obesity participants were those whose BMI ≥ 30 (16). Poor participants were whose ratio of family income to poverty <1. For the definition of drinking status, all participants needed to answer this question,” Had at least 12 alcohol drinks/1 yr?”. Those who answered “No” were labeled as “No drinking,” while those who answered “Yes” were labeled “drinking”. Waist circumference (WC) < 102 cm was considered normal for men, and WC≥ 102 cm was considered Aobesity. WC < 88 cm was considered normal for women, and WC ≥ 88 cm was considered Aobesity (17).

Hypertension was defined as a diagnosis of hypertension, the use of antihypertensive drugs, a systolic blood pressure ≥140 mm Hg, or a diastolic blood pressure ≥90 mm Hg. DM was defined as self-reported, diagnosed diabetes, glycohemoglobin≥ 6.5%, or fasting plasma glucose ≥126 mg/dL. Hyperlipidemia was defined as taking antihyperlipidemic drugs, TG ≥200 mg/dL, TC ≥150 mg/dL, HDL <40 mg/dL for males and <50 mg/dL for females, and LDL ≥130 mg/dL. Heart failure participants were those who were informed that they had heart failure. The cancer participants were those who had been told that they had cancer. Thyroid disease participants were those who were told that they had cancer-related thyroid disease, or thyroid antibody abnormalities were defined as TPOAb≥5.61 IU/ml or TGA≥4.11 IU/ml.

The Dietary Inflammatory Index (DII) is a credible measure of the extent to which dietary factors contribute to an individual’s inflammatory response (18). The DII score of night eating was calculated from the 24-h dietary recall data according to the calculation method described in the R package dietaryindex (19). In this study, we used total alcohol, beta-carotene, caffeine, carbohydrate, cholesterol, energy, fat, fiber, folic acid, vitamins (A, C, E, B1, B12), iron, zinc, magnesium, monounsaturated, polyunsaturated and saturated fatty acids, niacin, ALA (octadecatrienoic acid), EPA (alpha-linolenic acid), protein, selenium and zinc to calculate DII scores.

### 2.6 Statistical analyses

The complex survey design factors involved in the NHANES, including weights, clustering, and stratification, were all considered as recommended by the NCHS analytical guidelines. Data analysis was performed via R version 4.3.0. Weighted multiple linear regression model were applied to evaluate the associations of the frequency of night eating with thyroid function and sensitivity, including FT3, FT4, TT3, TT4, TSH, TG, TGA, TPOAb, FT3/FT4, TSHI, TT4RI and TFQI (no-night eating group as a reference), in three different model. The results are expressed as OR with 95% confidence intervals (CIs). We adjusted for no variable in Model 1. Model 1 is the unadjusted model. Model 2 was adjusted for baseline sex, age, BMI, race, marital status, family income and education. Model3 were further adjusted for baseline alcohol consumption, smoking status, physical activity, the DII of night eating, urinary iodine concentration, DM status, hypertension status, obesity status, hyperlipidemia status, abdominal obesity status and =UIC=. Owing to the presence of outliers in the data, some data were log-transformed, including T3, T4, TSH, Tg, TGA, TPOAb and TT4RI. We performed subgroup analyses with categorical variables, including sex, age, BMI, race, alcohol consumption, smoking status, physical activity, DII of night eating, UIC, DM, hypertension, obesity, hyperlipidemia, and Aobesity. We performed interaction term tests to check for heterogeneity between subgroups. Weighted multiple linear regression model were applied to investigate the correlations among the frequency of night eating and biochemical variables, including serum urea nitrogen (Ur), serum creatinine (Cr), HDL, LDL, TC, TG, and the neutrophil–lymphocyte ratio (NLR). The results are expressed as OR values with 95% CIs. Three model (model 1, 2, and 3) were adjusted as described above. A two-tailed p value of less than 0.05 was considered significant. The baseline characteristics are expressed as the means ±  Standard error of mean (SME) or numbers (percentages).

## 3 Results

### 3.1 Baseline characteristics of the participants

This study included 6093 participants from NHANES 2007–2012. The baseline characteristics of the participants across night eating frequencies are shown in **Table 1**. In terms of the frequency of night eating, compared with participants who did not eat at night, those who had more frequent night eating behavior were more likely to be young, be male, Non-Hispanic Black, single, and poor, have higher levels of education, like smoking, sleep less than 6 hours, be less likely to have hyperlipidemia, have higher DII levels of night eating, have higher FT3, have lower TSH, have higher Tg, higher FT3/FT4, lower TSHI, lower TT4FRI, higher Cr, lower glycohemoglobin, lower TC, lower LDL and lower HDL at baseline.

**Table 1:** Baseline characteristics of the participants.

	Overall (n=6093)	No night eating (n=4829)	One time (n=515)	Two times and over (n=522)	p value
Age					
18~34	2143(35.18)	1606(33.26)	215(41.81)	268(51.46)	<0.001
35~64	3332(54.68)	2693(55.77)	255(49.61)	241(46.14)	
≥65	618(10.14)	530(10.97)	44(8.57)	13(2.4)	
BMI					
<25	2068(33.94)	1593(32.98)	187(36.35)	204(39)	0.219
25~29	1694(27.8)	1371(28.4)	124(24.04)	130(24.96)	
≥30	2331(38.26)	1865(38.62)	204(39.61)	188(36.04)	
Gender					
Male	3313(54.38)	2556(52.93)	295(57.26)	335(64.09)	0.001
Female	2780(45.62)	2273(47.07)	220(42.74)	187(35.91)	
Race					
Mexican American	563(9.24)	465(9.62)	40(7.83)	39(7.43)	<0.001
Non-Hispanic White	3991(65.5)	3218(66.64)	329(63.88)	314(60.09)	
Non-Hispanic Black	737(12.1)	554(11.48)	72(14.07)	96(18.3)	
Other	801(13.15)	592(12.26)	73(14.22)	74(14.19)	
Marital					
Yes	3699(60.71)	3020(62.54)	270(52.47)	256(49.02)	<0.001
No	2394(39.29)	1809(37.46)	245(47.53)	266(50.98)	
PIR					
Poor	891(14.63)	662(13.71)	92(17.87)	96(18.4)	0.027
Not poor	5202(85.37)	4167(86.29)	423(82.13)	426(81.6)	
Education					
Below high school	388(6.37)	324(6.7)	24(4.65)	16(3.1)	0.005
High School	779(12.79)	621(12.85)	75(14.53)	55(10.58)	
Above high School	4926(80.84)	3885(80.45)	416(80.82)	451(86.31)	
Smoke					
Never	3437(56.41)	2782(57.6)	272(52.8)	257(49.25)	0.001
Former	1285(21.09)	1032(21.38)	108(20.95)	95(18.15)	
Now	1372(22.51)	1015(21.02)	135(26.26)	170(32.6)	
Drink					
Yes	4331(71.08)	3487(72.2)	379(73.64)	375(71.89)	0.842
No	1762(28.92)	1342(27.8)	136(26.36)	147(28.11)	
Activity					
No	3244(53.24)	2569(53.19)	296(57.45)	251(48.08)	0.053
Yes	2849(46.76)	2260(46.81)	219(42.55)	271(51.92)	
Sleep time					
<6 h	869(14.27)	647(13.39)	91(17.67)	98(18.72)	0.021
6~8 h	4848(79.56)	3880(80.35)	394(76.56)	390(74.77)	
>8 h	376(6.17)	302(6.26)	30(5.76)	34(6.51)	
Hypertension					
Yes	1869(30.67)	1501(31.09)	156(30.27)	138(26.41)	0.243
No	4224(69.33)	3328(68.91)	359(69.73)	384(73.59)	
Hyperlipidemia					
Yes	3892(63.87)	3129(64.8)	303(58.77)	305(58.35)	0.032
No	2201(36.13)	1700(35.2)	212(41.23)	217(41.65)	
Obesity					
Yes	1970(32.34)	1580(32.71)	181(35.14)	150(28.66)	0.169
No	4123(67.66)	3249(67.29)	334(64.86)	372(71.34)	
AObesity					
Yes	4132(67.81)	3330(68.96)	337(65.44)	327(62.55)	0.065
No	1961(32.19)	1499(31.04)	178(34.56)	195(37.45)	
DM					
Yes	598(9.81)	465(9.62)	60(11.58)	40(7.65)	0.269
No	5495(90.19)	4364(90.38)	455(88.42)	482(92.35)	
UIC					
Deficient	2020(33.15)	1624(33.62)	169(32.79)	167(32)	0.679
Normal	1960(32.17)	1572(32.55)	156(30.29)	163(31.27)	
Excessive	2113(34.68)	1634(33.83)	190(36.92)	192(36.73)	
**DII**	0.48 ± 0.02	0 ± 0	2.17 ± 0.09	3.62 ± 0.04	<0.001
**T3**	1.83 ± 0.01	1.83 ± 0.01	1.81 ± 0.02	1.83 ± 0.02	0.756
**T4**	7.74 ± 0.02	7.75 ± 0.02	7.64 ± 0.06	7.71 ± 0.07	0.195
**FT3**	3.23 ± 0.01	3.22 ± 0.01	3.26 ± 0.02	3.32 ± 0.02	<0.001
**FT4**	0.79 ± 0	0.79 ± 0	0.79 ± 0.01	0.79 ± 0.01	0.97
**TSH**	1.75 ± 0.01	1.75 ± 0.02	1.83 ± 0.07	1.64 ± 0.04	0.044
**TGA**	0.66 ± 0	0.67 ± 0.01	0.64 ± 0.01	0.65 ± 0.01	0.094
**Tg**	14.83 ± 0.45	14.81 ± 0.55	14.25 ± 0.67	15.74 ± 0.71	0.004
**TPOAb**	0.86 ± 0.01	0.88 ± 0.01	0.77 ± 0.03	0.77 ± 0.04	0.07
**FT3/FT4**	4.2 ± 0.01	4.19 ± 0.01	4.24 ± 0.03	4.31 ± 0.03	0.006
**TSHI**	1.76 ± 0.01	1.77 ± 0.01	1.78 ± 0.03	1.7 ± 0.03	0.041
**TT4RI**	173.36 ± 1.45	174.38 ± 1.59	179.02 ± 6.44	161.85 ± 4.48	0.008
**TFQI**	-0.06 ± 0	-0.07 ± 0	-0.06 ± 0.01	-0.07 ± 0.01	0.882
**Ur**	4.46 ± 0.02	4.49 ± 0.02	4.31 ± 0.07	4.29 ± 0.06	0.079
**Cr**	76.62 ± 0.28	76.56 ± 0.32	78.33 ± 0.92	77.2 ± 0.71	0.04
**Glycohemoglobin**	5.53 ± 0.01	5.53 ± 0.01	5.53 ± 0.04	5.49 ± 0.04	0.008
**TC**	5.04 ± 0.01	5.07 ± 0.02	4.94 ± 0.05	4.89 ± 0.05	0.002
**TG**	1.78 ± 0.02	1.74 ± 0.02	1.83 ± 0.07	1.9 ± 0.08	0.799
**LDL**	2.97 ± 0.01	2.99 ± 0.01	2.91 ± 0.04	2.79 ± 0.04	0.038
**HDL**	1.34 ± 0.01	1.35 ± 0.01	1.3 ± 0.02	1.3 ± 0.02	0.031
**NLR**	2.13 ± 0.01	2.12 ± 0.02	2.18 ± 0.05	2.06 ± 0.05	0.068

Continuous variables are presented as the means ± SEM, and p values were calculated via survey-weighted linear regression analysis. The categorical variables are presented as percentages, and p values were calculated via the survey-weighted chi-square test. BMI, body mass index; PIR, poverty income ratio; BMI, body mass index; Aobesity, abdominal obesity; DM, DM; UIC, urinary iodine concentration; DII, dietary Inflammatory Index of night eating; TT3, total triiodothyronine; TT4, total thyroxine; FT3, free triiodothyronine; FT4, free thyroxine; TSH, thyroid-stimulating hormone; Tg, thyroglobulin; TGA, thyroglobulin antibodies; TPOAb, thyroid peroxidase antibodies; FT3/FT4, FT3-FT4 ratio; TSHI, thyroid-stimulating hormone; TT4RI, thyrotroph thyroxine tesistance Index; TFQI, thyroid feedback quantile-based index; Ur, serum urea nitrogen; Cr, serum creatinine; TC, total cholesterol; TG, total triglycerides; LDL, low-density lipoprotein cholesterol; HDL, high-density lipoprotein cholesterol; NLR, neutrophil–lymphocyte ratio.

### 3.2 The association between frequency of night eating and thyroid function and thyroid hormone sensitivity

**Figure 2:**
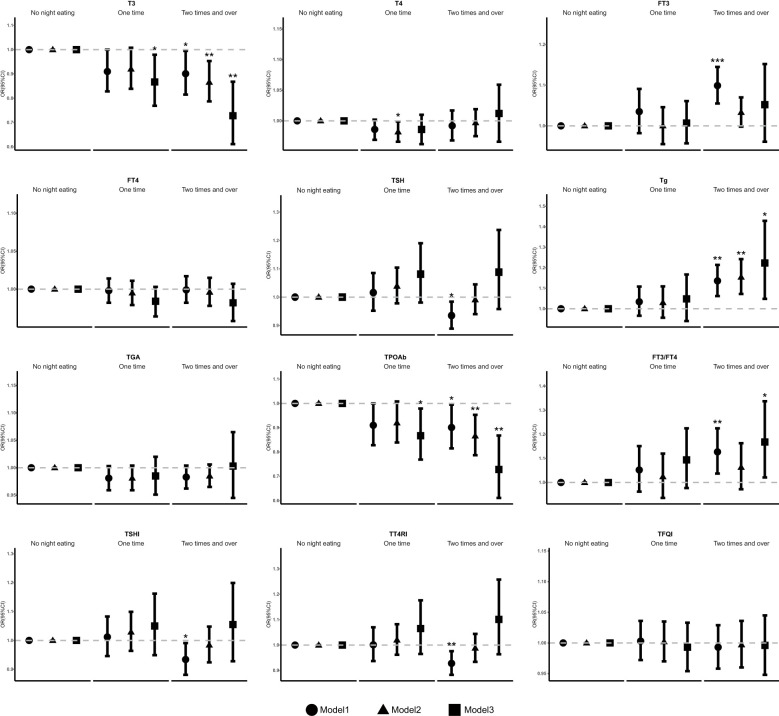
Association between the frequency of night eating and thyroid function and sensitivity. OR, odds ratio, Cl, confidence interval. * p < 0.05, ** p < 0.01, *** p < 0.001; p < 0.05 was considered statistically significant. Model: 1 unadjusted. Model 2: further adjusted for sex, age, BMI, race, marital status, education, and family income. Model 3: further adjusted for smoking behavior, physical activity, drinking behavior, sleep time, hypertension, hyperlipidemia, DM, UIC, and the Dill of night eating.

The association of frequency of night eating with thyroid function and thyroid hormone sensitivity were evaluated (**Figure 2**, **Supplementary Table S1**). Compared with people who had no night eating, individuals who had frequent night eating were negatively associated with T3 levels in model 1, which was adjusted for nothing (OR 0.901 [95% CI 0.815, 0.995], p trend=0.104]). This association remained significant after further adjusting for sex, age, BMI, race, marital status, education, and family income in model 2 (OR 0.866 [95% CI 0.787, 0.953], p trend=0.064). Model 3, which was further adjusted for smoking behavior, physical activity, drinking behavior, sleep time, hypertension, hyperlipidemia, DM, UIC, and the DII of night eating, remained significant (OR 0.728 [95% CI 0.611, 0.868], p trend=0.235). For TPOAb, the results showed a similar trend to that at T3. In model 1, the OR was 0.901 (95% CI 0.815, 0.995), with a p trend of 0.104. In model 2, the OR was 0.866 (95% CI 0.787, 0.953), with a p trend of 0.064. In model 3, the OR was 0.728 (95% CI 0.611, 0.868), with a p trend of 0.235. Conversely, there was a positive association with Tg levels in model 1 (OR 1.36 [95% CI 1.026, 1.214], p trend=0.047), model 2 (OR 1.154 [95% CI 1.072, 1.242], p trend=0.088) and model3 (OR 1.223 [95% CI 1.048, 1.429], p trend=0.015).

Compared with people who had no night eating, individuals who had more frequent of night eating were positively associated with FT3 (OR 1.099 [95% CI 1.055, 1.145], p trend=0.011) and negatively associated with TSH (OR 0.935 [95% CI 0.889, 0.984], p trend=0.025) in model 1. Moreover, compared with people who had no night eating, individuals who consumed one time at night were negatively associated with T3 (OR 0.867 [95% CI 0.769, 0.979], p trend=0.235) in model 3 and T4 (OR 0.982 [95% CI 0.966, 0.999], p trend=0.266) in model 2.

Further analyses of thyroid sensitivity revealed that, compared with no night eating, more frequent night eating behavior were positively associated with FT3/FT4 levels in model 1 (OR 1.127 [95% CI 1.037, 1.225], p trend=0.05) and model3 (OR 1.168 [95% CI 1.021, 1.337], p trend=0.516), but this association was not statistically significant in model 2. Conversely, a significantly negative association with the TSHI (OR 0.934 [95% CI 0.881, 0.991], p trend=0.051) and TT4RI (OR 0.928 [95% CI 0.883, 0.976], p trend=0.015) was detected in model 1, but no statistically significant association was detected in the other model.

### 3.3 The association of frequency of night eating and other laboratory biochemical variables

**Figure 3:**
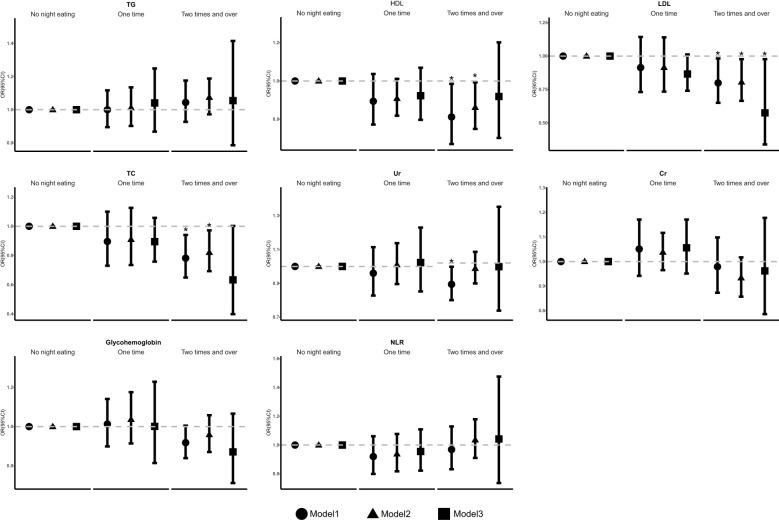
The association of frequency of night eating and other laboratory biochemical variables. OR, odds ratio. Cl, confidence interval. * p < 0.05, ** p < 0.01, *** p < 0.001; p < 0.05 was considered statistically significant. Model 1: unadjusted. Model 2. further adjusted for sex, age, BMI, race, marital status, education, and family income. Model 3: further adjusted for smoking behavior, physical activity, drinking behavior, sleep time, hypertension, hyperlipidemia, DM, UIC, and the Dll of night eating.

Furthermore, weighted linear regression analysis was applied to investigate the relationships between the frequency of night eating and other biochemical variables (**Figure 3**). Statistical differences were found in laboratory tests for blood lipids between those who had higher eating frequency at night and those who did not. Our results indicate that, compared with people who did not eat at night, individuals who consumed at least one night were negatively associated with HDL levels in model 1 (OR 0.812 [95% CI 0.670, 0.985]) and model 2 (OR 0.862 [95% CI 0.749, 0.993]). Moreover, it was also negatively associated with LDL levels in model 1 (OR 0.799 [95% CI 0.650, 0.982]), model 2 (OR 0.806 [95% CI 0.665, 0.977]) and model 3 (OR 0.575 [0.339, 0.977]) and with TC levels in model 1 (OR 0.782 [95% CI 0.649, 0.941]) and model 2 (OR 0.821 [95% CI 0.693, 0.972]) and with Ur levels in model 1(OR 0.874 [95% CI 0.780, 0.978]). The laboratory parameters Cr, glycohemoglobin, NLR, and TG were not significantly different among the model.

### 3.4 Subgroup analysis

**Figure 4:**
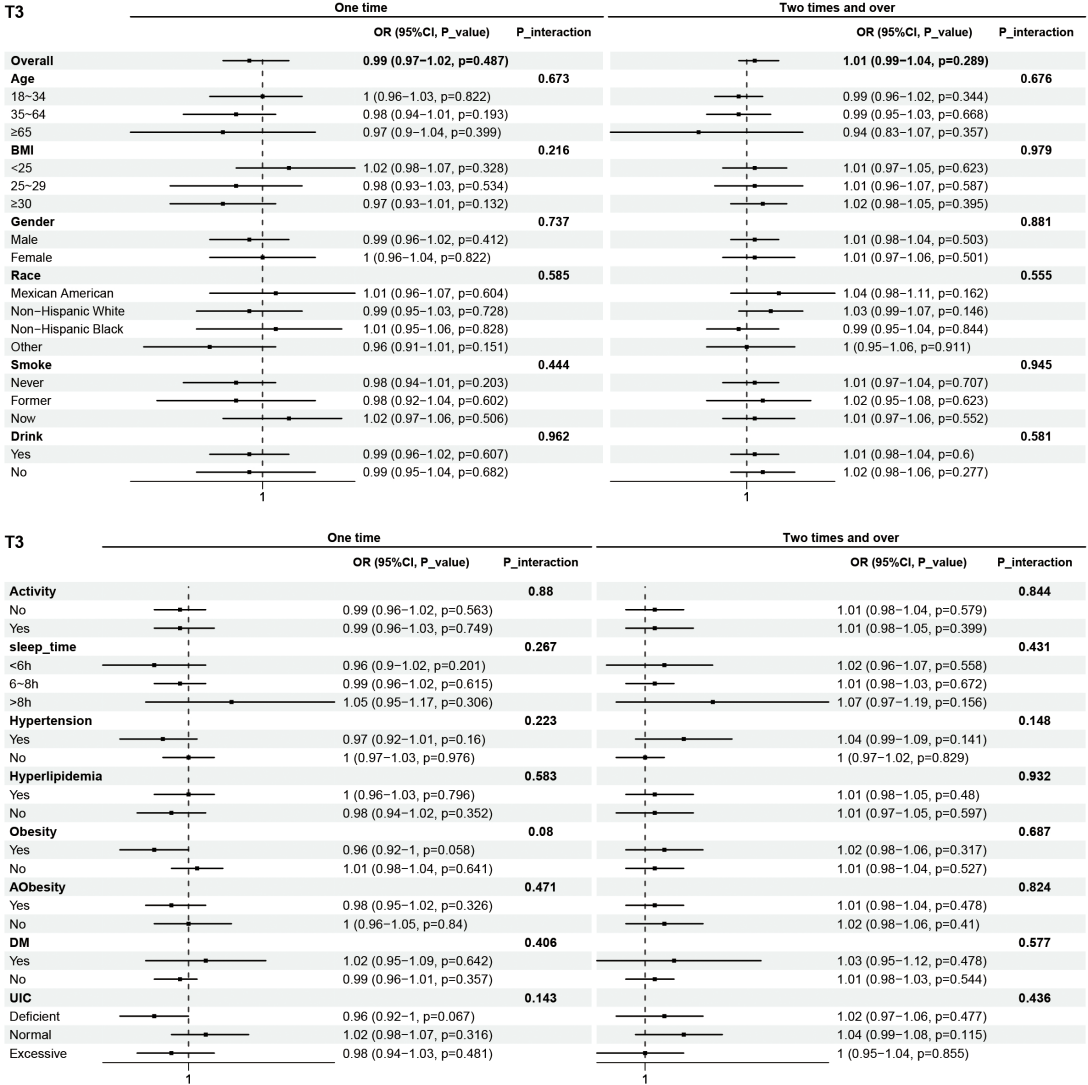
Subgroup analysis of association between night eating frequency and T3 levels. The "No night eating" group was used as a reference.

**Figure 5:**
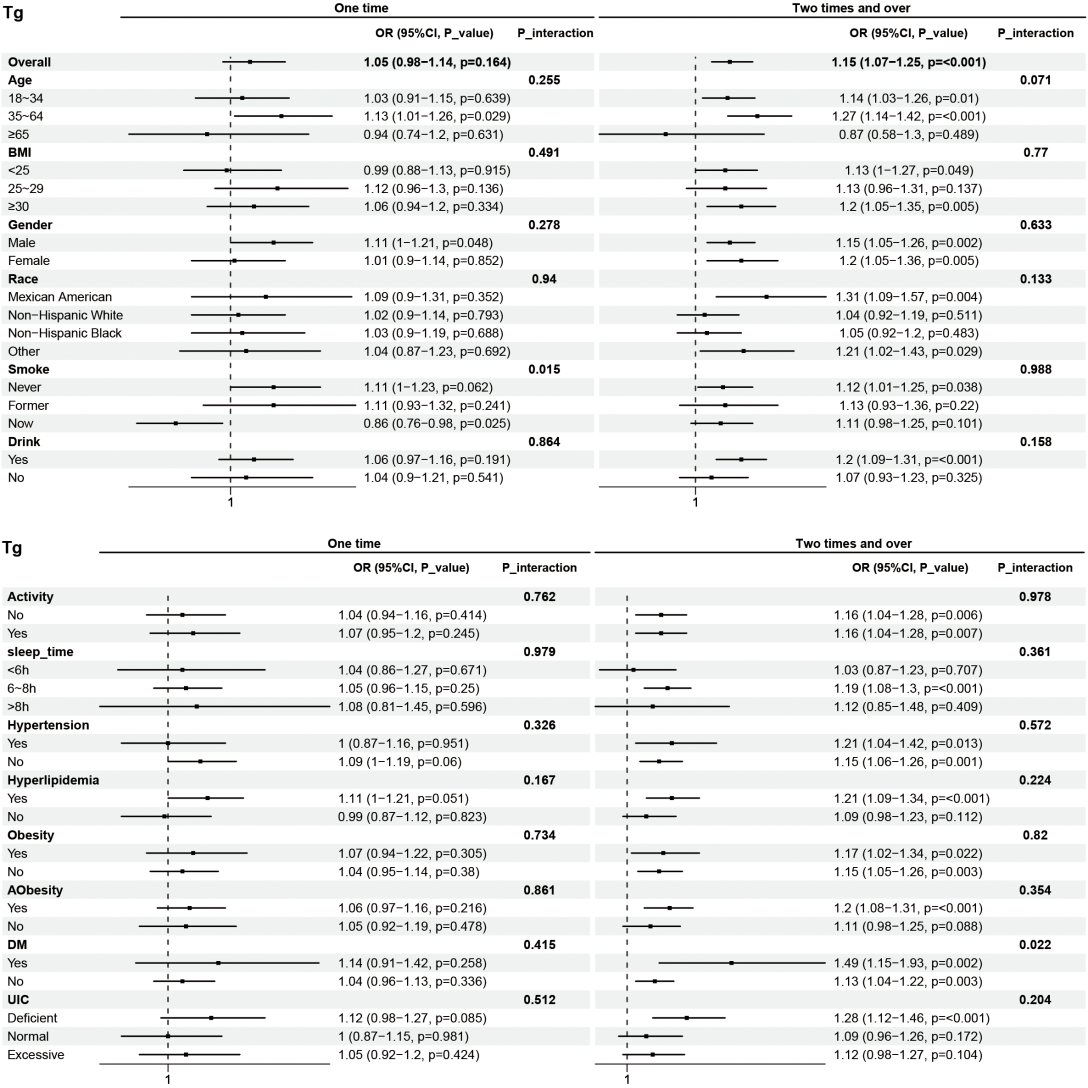
Subgroup analysis of association between night eating frequency and Tg levels. The "No night eating" group was used as a reference.

**Figure 6:**
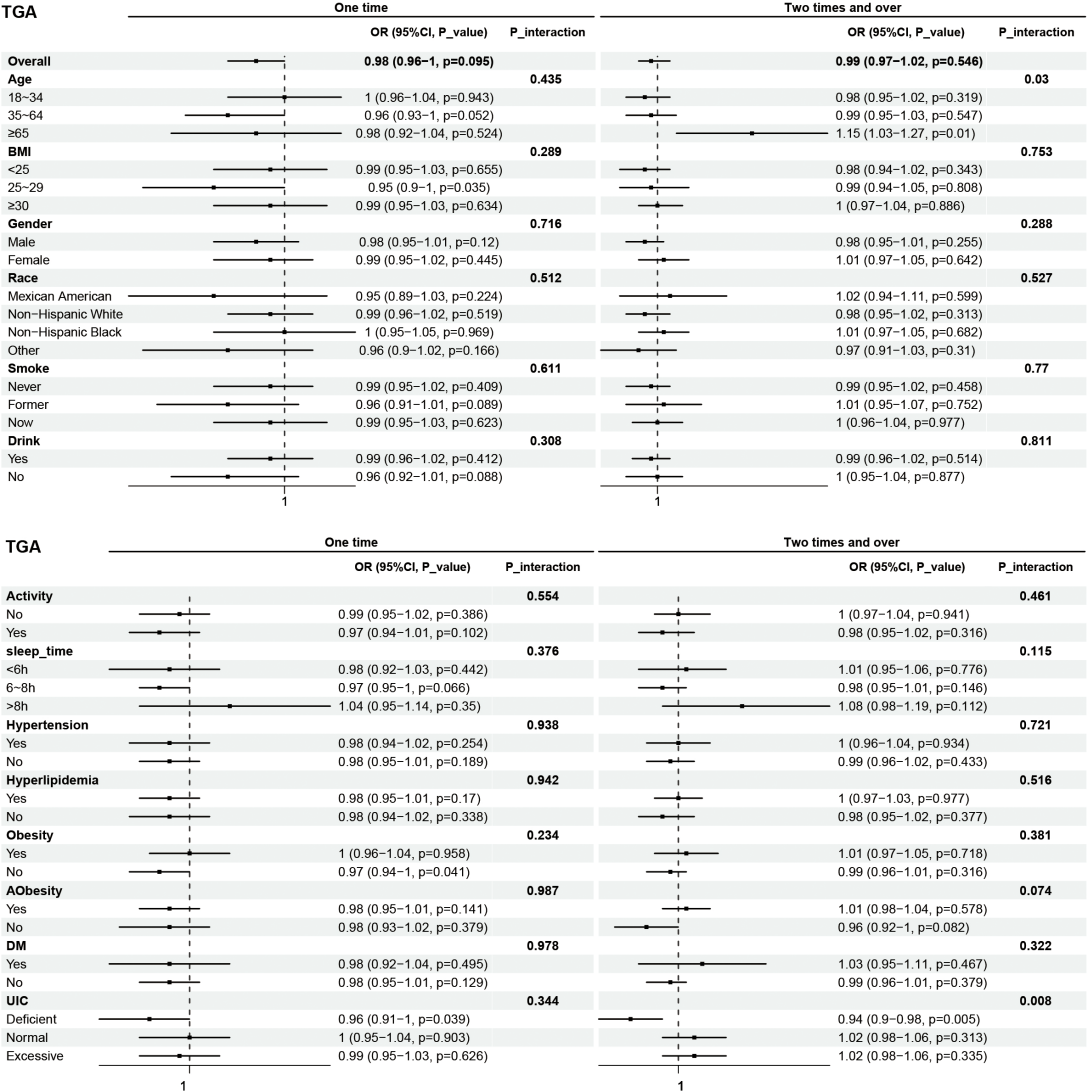
Subgroup analysis of association between night eating frequency and TGA levels. The "No night eating" group was used as a reference.

**Figure 7:**
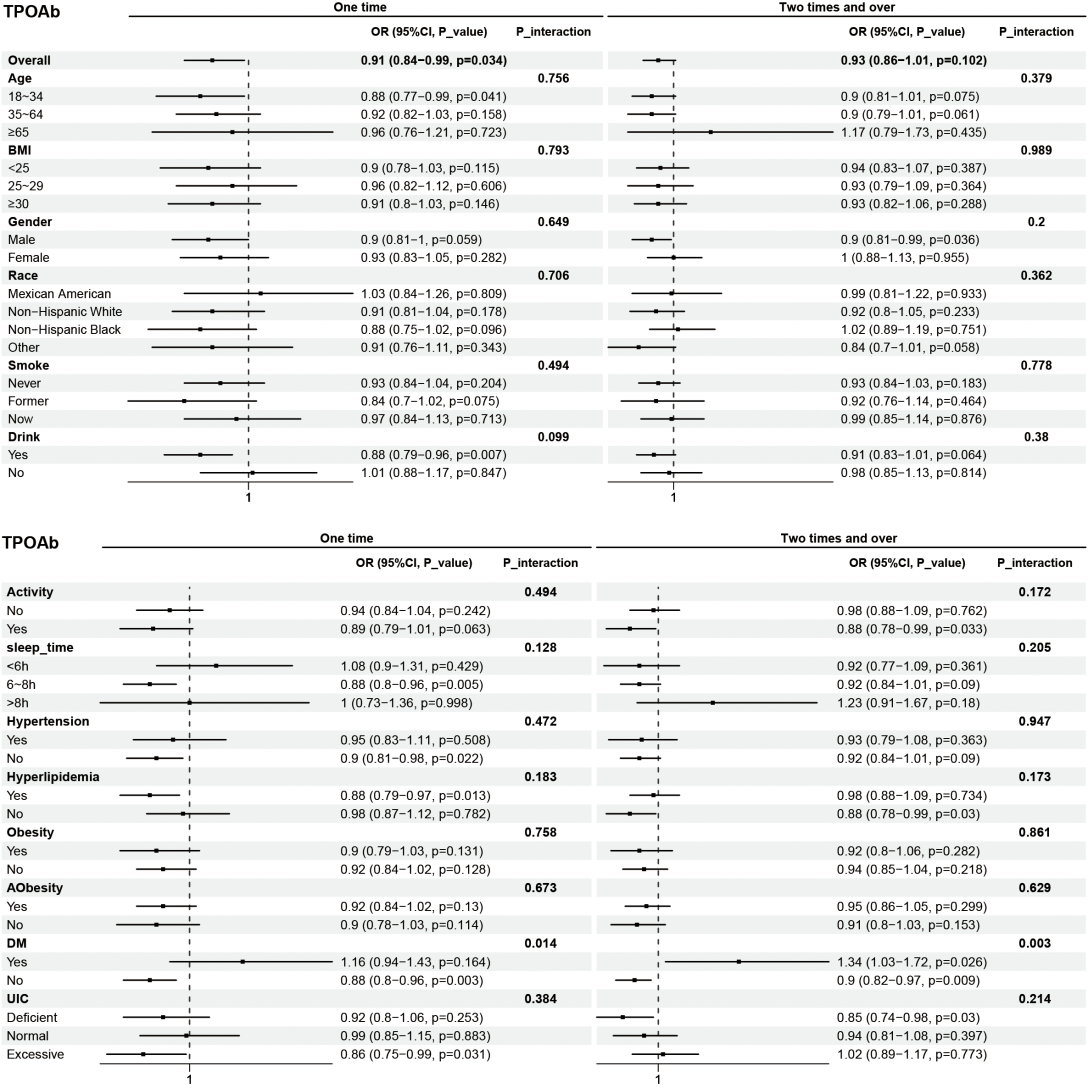
Subgroup analysis of association between night eating frequency and TPOAb levels. The "No night eating" group was used as a reference.

Subgroup analyses were performed to examine the associations between eating frequency and T3, Tg, TGA, and TPOAb levels in various populations that were categorized according to age, BMI, sex, race, smoking status, alcohol consumption, physical activity, sleep time, hypertension, hyperlipidimia, obesity, Aobesity, DM, and UIC (**Figures 4**–**7**). The results revealed that none of the subgroups mentioned above affected the association between the frequency of night eating and T3 (each p interaction > 0.05). For Tg levels, it is noteworthy that a significant positive association between the frequency of night eating and the number of participants with (OR 1.49 [95% CI 1.15, 1.93]), p interaction=0.022) or without (OR 1.13 [95% CI 1.04, 1.22]) DM was found. Moreover, an interaction effect between smoking and one time night eating on Tg levels was also found (OR 0.86 [95% CI 0.79, 0.98]), p interaction=0.025). For TGA levels, a significant positive association with higher eating frequency at night was found in participants aged 65 years and older (OR 1.15 [95% CI 1.03, 1.27]), p interaction=0.03). Conversely, a significant negative association with higher eating frequency at night eating was found in the participants with UIC (OR 0.94 [95% CI 0.9, 0.98]), p interaction=0.008). Furthermore, TPOAb was first found to be negatively associated with one-time (OR 0.88 [95% CI 0.8, 0.96]), p interaction=0.014) or more-time (OR 0.9 [95% CI 0.82, 0.97]), p interaction=0.003) night eating in participants without DM. Conversely, a significant positive association with more night eating was found in the participants with DM (OR 1.34 [95% CI 1.03, 1.72]), p interaction=0.003).

## 4 Discussion

This study expands on existing research regarding night eating frequency and is the first, to our knowledge, to examine its relationship with thyroid function and sensitivity. To investigate this association, we analyzed data from a nationally representative cohort of U.S. adults. To reduce potential confounding factors, we excluded participants who were under 18 years of age, had cancer, had heart failure, were pregnant, or had thyroid disease. Our findings revealed a strong correlation between thyroid function indices and night eating frequency. Specifically, more frequent night eating was associated with higher levels of Tg and lower levels of T3 and TPOAb. Additionally, our study revealed that, compared with not eating at night, more frequent night eating was linked to lower LDL levels. Subgroup analysis, stratified by DM status, indicated that among participants with DM, increased night eating frequency might lead to more pronounced increases in Tg and TPOAb levels. Conversely, in healthy participants, frequent night eating was associated with a decrease in TPOAb levels. Notably, we observed that among participants aged 65 years and older, frequent night eating led to a relative increase in TGA levels, whereas the opposite effect was observed in those with UIC deficiency. Importantly, our results did not reveal a statistically significant difference between thyroid sensitivity and night eating frequency.

Regulating the timing of food intake throughout the day can influence the rhythmicity and regularity of certain aspects of the circadian system and related behaviors (20). Organisms encounter different rhythmic occurrences, such as daily and seasonal cycles. To effectively cope with these cyclic changes in their environment, organisms have developed biological clocks. Almost every cell in the body possesses an intrinsic circadian clock lasting approximately 24 hours, which meticulously governs physiological processes and hormonal rhythms (21). Studies on rodents have shown that consuming a high-fat diet during the rest phase (the light phase for nocturnal rodents) leads to more significant weight gain than does consuming the same diet during the active phase (22). Furthermore, nocturnal light exposure increases body weight by altering the timing of food intake (23). In addition, many previous studies have suggested that disruptions in circadian rhythms may affect the endocrine system, potentially leading to obesity, DM and cardiovascular disease (24–26). The hypothalamus-pituitary-thyroid axis is regulated by the circadian rhythm through the central circadian pacemaker located in the suprachiasmatic nuclei of the anterior hypothalamus (27). Moreover, the literature has reported that the expression profiles of circadian clock genes are abnormal in well-differentiated thyroid cancer but not in benign nodules or healthy thyroids (14). Disruptions in circadian rhythms have also been associated with elevated TSH levels, increasing the risk of thyroid nodules and autoimmune thyroiditis (AIT) (14, 26, 28). AIT, also known as Hashimoto’s thyroiditis (HT) or chronic lymphocytic thyroiditis, is characterized by excessive production of TGA and TPO and infiltration of lymphocytes into thyroid tissue. Notably, a previous study demonstrated a broad disruption of clock gene profiles within thyroid samples from patients with autoimmune thyroiditis (28). Interestingly, numerous studies have reported the effects of Ramadan fasting on thyroid function in Muslim populations. During Ramadan, Muslims are required to abstain from food and drink between dawn and sunset as part of their religious obligations. This practice not only disrupts eating patterns and sleep schedules but also disturbs circadian rhythms. Current research indicates that in healthy adults, TSH levels increase significantly throughout the fasting month, while fT4 and fT3 levels remain unchanged (29). However, in individuals with hypothyroidism, Ramadan fasting worsens thyroid function (30, 31). Shift workers, whose circadian rhythms are significantly disrupted, also exhibit variations in thyroid hormone levels under different dietary influences (32). This suggests a dual impact of nighttime eating and circadian rhythm disturbances on thyroid function. Our study revealed that more frequent night eating resulted in a decrease in T3 and TPOAb levels and an increase in Tg levels, but TSH concentrations were not significantly different from no night eating participants, which suggested a complex interaction between circadian rhythms and thyroid dysfunction.

Importantly, we evaluated the night eating DII. The DII, proposed by J.R. Hébert et al. (33), is a literature-derived dietary tool for measuring individual dietary inflammation. Many studies have shown that higher increased levels of IL-6 and CRP (34–36), which results in increased FT3 and TT4 levels (37, 38) and increased risk of thyroid carcinoma (39). Our results revealed that those who ate two or more times at night had higher DII scores. This may be because multiple eating episodes resulted in a higher inflammation score. Therefore, we speculate that in addition to rhythm disruption, dietary inflammation may also be a contributing factor. Specifically, we hypothesize that when diurnal eating rhythms are disrupted and night eating promotes inflammation, thyroid function may be more severely impaired.

Thyroid hormones are reportedly related to pancreatic β-cell development and influence glucose metabolism through several organs (38). Therefore, thyroid disease and DM are two closely associated disorders. Compared with the general population, type 1 diabetes(T1DM) patients develop thyroid disease at an early age (40). The prevalence of thyroid disease is significantly greater among patients with type 2 diabetes (T2DM) than in the general population (41). In individuals with DM and normal thyroid function, the nocturnal TSH peak has been found to be absent or weak, and the TSH response to TRH is also impaired (42). Although the prevalence of thyroid dysfunction was greater in the T2DM group, TPOAb and TGA were more common in the healthy group (43). In addition, a study reported a greater prevalence of thyroid disease in subjects in the United States with DM than in those without DM, especially in patients who were TPOAb positive. Surprisingly, our results revealed that frequent night eating may be one of the reasons for the increased production of TPOAb in diabetic patients, even if the effects of glucose metabolism disorders are not as significant as those caused by circadian rhythm disruptions.

A higher prevalence of thyroid disorders has been documented in people with DM than in normoglycemic individuals, whereas patients with both endocrinopathies have poorer glycemic control and are more vulnerable to the development of complications. We speculate that the reason for the elevated Tg levels may be that night eating caused an increase in blood glucose levels, prompting the secretion of thyroid hormones to participate in glucose homeostasis, which also positively affected the Tg level. Therefore, owing to the interaction effects, the Tg levels in DM participants increased more than those in participants who did not eat at night.

As we all know, iodine intake significantly affects thyroid function. Chronic exposure to excess iodine intake induces autoimmune thyroiditis, partly because highly iodinated Tg is more immunogenic (44). Conversely, iodine deficiency may decrease the level of iodinated Tg and lead to a decrease in TGA levels. This situation is more pronounced in participants with iodine deficiency, who eat more times at night. Furthermore, our results indicate that the elevation in Tg levels is more pronounced in the elderly population, particularly those who eat more times at night. One study reported that the level of TGA gradually increased with increasing age in females, whereas TGA levels peaked in males aged 50–59 years (42), which is consistent with our results.

Additionally, we focused on laboratory indicators such as renal function, glycated hemoglobin, blood lipids, and the NLR, in addition to thyroid function, with the intention of identifying any associations between these indicators and impaired thyroid function. Thyroid hormones are known to affect energy metabolism. Many patients with metabolic syndrome have subclinical or clinical hypothyroidism and vice versa (45). A study of railway workers revealed that irregular work schedules, which affect their circadian rhythm of sleep, may lead to dyslipidemia (46). Another study indicated that men who slept less than 7 h had a greater probability of dyslipidemia than day workers did (47). One study reported that subjects consumed a specified snack (192.4 ± 18.3 kcal) either during the10:00AMor 23:00PM for 13 days. Compared with the daytime group, the night eating group presented increased LDL levels (12). In addition, the results of a meta-analysis indicated that increased TSH levels were significantly associated with increased TC and LDL levels (48). The metabolically unhealthy no-obese (MUNO) phenotype is significantly associated with hypothyroidism in individuals with thyroid autoimmunity, with a pronounced prevalence in women (49). The above studies revealed the intricate relationships among lipid metabolism, thyroid hormones, and the circadian rhythm. Notably, our results revealed that more frequent night eating was associated with lower levels of LDL, and these findings are almost consistent with those reported in another study (50). However, in the subgroup analyses of obesity, Aobesity, BMI, and hyperlipidemia, no interactions were found between these indicators and T3, Tg, TPOAb, or TGA. The common perception is that circadian rhythm disruptions lead to an increase in LDL, typically regarded as a “bad” lipid parameter, which in turn increases the risk of developing cardiovascular diseases and disrupts thyroid hormone secretion (51). However, our research revealed the opposite findings. These results, not only stem from variations in study methods, subjects, and populations, but also suggest the presence of potential underlying mechanisms that warrant further investigation.

Our study also had some limitations. First, the participants included in the study did not represent the entire population. Second, the frequency of night eating was obtained through dietary recall, the accuracy of which cannot be reliably estimated. Additionally, night eating information is only a single dietary recall and cannot represent long-term habits. Third, unknown and unmeasured confounding factors are likely present, so we cannot make strong causal inferences. Fourth, we could not determine whether participants were taking any medications or supplementation. Given the limitations mentioned above, the present results still need further confirmation by longitudinal prospective large cohort studies with accurate information.

## Data Availability

Publicly available datasets were analyzed in this study. This data can be found here: https://wwwn.cdc.gov/nchs/nhanes/Default.aspx.
