# Supplementary material for: Association between night eating frequency and thyroid function and sensitivity: a cross-sectional study from the NHANES database
Source: Front Endocrinol (Lausanne). 2024 Dec 23;15:1489459. doi: 10.3389/fendo.2024.1489459 (PMC11700809; doi:10.3389/fendo.2024.1489459)
Supplement: Supplementary file 1 [file Table1.docx]

**Supplementary Table 1** The association of frequency of night eating with thyroid function and thyroid hormone sensitivity

| **Thyroid function and sensitivity** | **Night eating frequency** | | | ***P* trend** |
| --- | --- | --- | --- | --- |
|  | **No night eating** | **One time** | **Two times or over** |  |
| **T3** |  |  |  |  |
| Model 1 | 1(1, 1) | 0.91(0.828, 1) | 0.901(0.815, 0.995) | 0.104 |
| Model 2 | 1(1, 1) | 0.92(0.839, 1.008) | 0.866(0.787, 0.953) | 0.064 |
| Model 3 | 1(1, 1) | 0.867(0.769, 0.979) | 0.728(0.611, 0.868) | 0.235 |
| **T4** |  |  |  |  |
| Model 1 | 1(1, 1) | 0.986(0.969, 1.002) | 0.992(0.968, 1.017) | 0.395 |
| Model 2 | 1(1, 1) | 0.982(0.966, 0.999) | 0.997(0.975, 1.019) | 0.266 |
| Model 3 | 1(1, 1) | 0.986(0.962, 1.01) | 1.012(0.966, 1.059) | 0.467 |
| **FT3** |  |  |  |  |
| Model 1 | 1(1, 1) | 1.035(0.982, 1.091) | 1.099(1.055, 1.145) | 0.011 |
| Model 2 | 1(1, 1) | 1(0.955, 1.046) | 1.033(0.998, 1.07) | 0.313 |
| Model 3 | 1(1, 1) | 1.007(0.957, 1.061) | 1.052(0.961, 1.152) | 0.995 |
| **FT4** |  |  |  |  |
| Model 1 | 1(1, 1) | 0.998(0.982, 1.014) | 0.999(0.982, 1.017) | 0.953 |
| Model 2 | 1(1, 1) | 0.995(0.979, 1.011) | 0.996(0.978, 1.015) | 0.694 |
| Model 3 | 1(1, 1) | 0.984(0.964, 1.003) | 0.982(0.958, 1.007) | 0.706 |
| **TSH** |  |  |  |  |
| Model 1 | 1(1, 1) | 1.016(0.952, 1.085) | 0.935(0.889, 0.984) | 0.025 |
| Model 2 | 1(1, 1) | 1.039(0.978, 1.104) | 0.991(0.94, 1.045) | 0.809 |
| Model 3 | 1(1, 1) | 1.081(0.981, 1.19) | 1.088(0.958, 1.237) | 0.561 |
| **Tg** |  |  |  |  |
| Model 1 | 1(1, 1) | 1.034(0.966, 1.108) | 1.136(1.062, 1.214) | 0.047 |
| Model 2 | 1(1, 1) | 1.03(0.956, 1.109) | 1.154(1.072, 1.242) | 0.088 |
| Model 3 | 1(1, 1) | 1.048(0.94, 1.167) | 1.223(1.048, 1.429) | 0.015 |
| **TGA** |  |  |  |  |
| Model 1 | 1(1, 1) | 0.981(0.959, 1.003) | 0.983(0.962, 1.004) | 0.655 |
| Model 2 | 1(1, 1) | 0.981(0.959, 1.004) | 0.985(0.965, 1.006) | 0.651 |
| Model 3 | 1(1, 1) | 0.985(0.951, 1.02) | 1.003(0.945, 1.065) | 0.183 |
| **TPOAb** |  |  |  |  |
| Model 1 | 1(1, 1) | 0.91(0.828, 1) | 0.901(0.815, 0.995) | 0.104 |
| Model 2 | 1(1, 1) | 0.92(0.839, 1.008) | 0.866(0.787, 0.953) | 0.064 |
| Model 3 | 1(1, 1) | 0.867(0.769, 0.979) | 0.728(0.611, 0.868) | 0.235 |
| **FT3/FT4** |  |  |  |  |
| Model 1 | 1(1, 1) | 1.052(0.962, 1.151) | 1.127(1.037, 1.225) | 0.050 |
| Model 2 | 1(1, 1) | 1.024(0.936, 1.12) | 1.063(0.972, 1.163) | 0.326 |
| Model 3 | 1(1, 1) | 1.094(0.977, 1.225) | 1.168(1.021, 1.337) | 0.516 |
| **TSHI** |  |  |  |  |
| Model 1 | 1(1, 1) | 1.012(0.946, 1.083) | 0.934(0.881, 0.991) | 0.051 |
| Model 2 | 1(1, 1) | 1.029(0.964, 1.099) | 0.984(0.924, 1.048) | 0.706 |
| Model 3 | 1(1, 1) | 1.05(0.949, 1.162) | 1.055(0.928, 1.199) | 0.698 |
| **TT4RI** |  |  |  |  |
| Model 1 | 1(1, 1) | 1.001(0.937, 1.07) | 0.928(0.883, 0.976) | 0.015 |
| Model 2 | 1(1, 1) | 1.02(0.962, 1.082) | 0.988(0.934, 1.044) | 0.534 |
| Model 3 | 1(1, 1) | 1.065(0.965, 1.176) | 1.101(0.964, 1.258) | 0.714 |
| **TFQI** |  |  |  |  |
| Model 1 | 1(1, 1) | 1.003(0.972, 1.036) | 0.993(0.958, 1.029) | 0.635 |
| Model 2 | 1(1, 1) | 1.002(0.97, 1.035) | 0.997(0.96, 1.036) | 0.785 |
| Model 3 | 1(1, 1) | 0.993(0.954, 1.033) | 0.996(0.948, 1.045) | 0.598 |
